# Mentoring for motivation: supporting student teachers’ basic psychological needs in Chinese public kindergarten internships

**DOI:** 10.3389/fpsyg.2025.1676131

**Published:** 2025-09-30

**Authors:** Yan Yang, Yingxin Chen

**Affiliations:** ^1^School of Education, Shanghai Normal University Tianhua College, Shanghai, China; ^2^Department of Education and Arts, Taishan Polytechnic, Tai'an, China

**Keywords:** psychological needs, intrinsic motivation, identity formation, early childhood student teachers, mentoring, teaching internship

## Abstract

This qualitative case study investigates how early childhood student teachers experience and interpret mentor support for their psychological needs of autonomy, competence, and relatedness during teaching internships in Chinese public kindergartens. Guided by self-determination theory, data were collected through semi-structured interviews with nine senior student teachers and analyzed using thematic analysis. The findings revealed that mentors foster student teachers’ psychological needs satisfaction through the following three key mechanisms: progressive autonomy fulfillment through selective empowerment, delegated instructional responsibility and personalized work assignment, gradual competence development via constructive feedback and optimal challenge in scaffolded tasks, and dual relatedness construction through professional collaboration and emotional care. The mechanisms enhance student teachers’ intrinsic motivation, engagement and professional identity formation. The study proposes the Developmentally Responsive Mentoring for Motivation (DRMM) model, which conceptualizes mentoring as a calibrated process of assessing readiness, assigning optimally challenging tasks, analyzing performance, and adjusting guidance, ensuring that support remains responsive to developmental and contextual needs. The model extends the applicability of self-determination theory and offers a transferable framework for teacher education programs.

## Introduction

Teaching internships have a unique role in the professional development of early childhood student teachers by integrating their theoretical training with practical teaching experience in a school setting, while simultaneously shaping their professional identities (Caires et al., 2012; Seyri and Nazari, 2023). These student teachers face specific challenges with maintaining a balance between compliance and innovation, applying theory to practice, and navigating relationships in work environment (Bloomfield, 2010). Moreover, student teachers frequently confront motivational and psychological impediments as they navigate unfamiliar classroom environment and complex mentoring dynamics, inhibiting their sense of autonomy, competence, and connection (Dreer, 2020; Huu Nghia and Tai, 2019; Kaplan, 2021). Effective mentorship, characterized by emotional support and a positive relationship, has been shown to enhance student teachers’ motivation and psychological well-being while lack of such support can increase vulnerability to feelings of inadequacy and isolation (Vansteenkiste et al., 2020). The quality of mentoring is therefore critical to their successful adaptation and growth (Schechter and Orly, 2014). Student teachers may establish different levels of relationship with mentors (i.e., mentorship). Mentorship featured by mutual respect, trust, and commitment to growth supports knowledge transfer, skill enhancement, and emotional well-being (Burgess et al., 2018; Lee and Chiang, 2020).

Understanding how student teachers are supported in their early professional development requires a theoretical lens that highlights intrinsic motivation and psychological functioning. The self-determination theory (SDT) offers such a perspective by focusing on autonomy, competence, and relatedness as basic psychological needs (Ryan and Deci, 2017). Fulfillment of these needs enhances intrinsic motivation and job satisfaction, which is especially pertinent to the growth of beginning teachers (Kaplan, 2022). For early childhood student teachers, fulfilling these needs during teaching practice aids their engagement, professional identity formation and sustainable professional growth (Hu et al., 2023; Jones et al., 2020).

In China, early childhood education reforms prioritize teacher quality. As part of this effort, teaching internships are primarily arranged in public kindergartens, which are considered more capable of providing structured mentoring to ensure standardized and supervised teaching practice. These kindergartens are organized into a pyramid system, ranging from model kindergartens to second-grade kindergartens. While public kindergartens are now central to internship arrangements in China, how mentorship within these settings supports student teachers’ basic psychological needs remains underexplored. Mentoring literature in China has mainly focused on the primary and secondary education, which leaves a scarcity of literature on mentoring within the early childhood education (Liu and Sitoe, 2019). In addition, the existing research relied heavily on quantitative surveys, limiting in-depth understanding of the nuanced and situated realities of student teachers in the context of early childhood internships in China (Linch et al., 2020). Moreover, literature pertaining to application of SDT in Chinese early childhood teacher education contexts is lacking (Yu et al., 2018). This study addresses these gaps by exploring how early childhood student teachers experience and interpret mentor support for their psychological needs of autonomy, competence, and relatedness during teaching internships. Bridging these gaps also requires consideration of culturally specific factors. Mentoring in China are influenced by the hierarchical mentor-mentee dynamics and collectivist culture, which emphasize respect for authority and harmony within groups (Lee et al., 2018). While these cultural dynamics might constrain autonomy, but they can simultaneously strengthen competence and relatedness through guidance and shared goals (Hagger and Protogerou, 2018). Applying SDT within this collectivist and authority-oriented context illustrates that autonomy, competence, and relatedness are culturally negotiated experiences (Wang et al., 2019). In China, early childhood teaching internships are guided by a structured framework involving two core stakeholders: mentors and student teachers (Ministry of Education of the People’s Republic of China, 2016). This staged framework aims to foster student teachers’ teaching autonomy as well as their professional growth. Initially, student teachers perform prescriptive duties like assisting with daily routines, observing children, and following mentors’ instructions to get familiar with classroom standards and professional expectations. As the internship proceeds, mentors gradually allow student teachers to independently organize small-group or half-day activities. At the final stage, student teachers are given full control over lesson planning and activity implementation. Through qualitative analysis of student teachers’ lived experiences, the research extends the cultural applicability of SDT and contributes to the discourse on early childhood teacher education reform in China. It highlights the importance of developmentally responsive mentorship in fostering student teachers’ sustained professional growth and career commitment.

## Literature review

### Theoretical framework

SDT explains how social contextual factors can support or hinder the development of psychological health by influencing the fulfillment of individuals’ basic psychological needs, and proposes three core basic psychological needs that are conducive to the positive development of individuals, namely, autonomy, competence, and relatedness. The autonomy need refers to the individual’s feeling that his or her behavior is in accordance with his or her own will, a need for self-discipline and self-governance; the competence need refers to the individual’s feeling that he or she can apply knowledge and has the ability to thus solve problems and overcome difficulties, a need for mastery of knowledge and competence; and the relatedness need refers to the individual’s feeling that he or she has established a good social relationship with other people, a need to establish a connection with other people (Ryan and Deci, 2017). The theory suggests that individual motivational development is a process from lack of motivation to external motivation to internal motivation, and that the human organism has an inherent tendency to develop intrinsic motivation, which actively develops towards natural development through the process of integration, and that the fulfillment of the three basic psychological needs will contribute to the development of internal motivation (Deci and Ryan, 2012).

The theory accordingly proposes six mini-theories: cognitive evaluation theory (CET), Organismic integration theory (OIT), causality orientations theory (COT), basic psychological needs theory (BPNT), goal contents theory (GCT), and relationship motivation theory (RMT), with the basic psychological needs theory focusing on explaining how the dynamic fulfillment of an individual’s basic psychological needs affects his or her sense of well-being and vitality, which posits that all individuals have basic needs for autonomy, competence, and relatedness (Ryan and Deci, 2017). The three needs are interdependent, and the fulfillment of each facilitates the fulfillment of the others. In different cultures, in different environments, and in different social relationships, when an individual’s three basic psychological needs are met, it promotes their positive development, such as individual vitality, well-being, and autonomy motivation, and conversely, it hinders the development of all aspects of the individual’s development and produces negative effects, such as depression and lack of autonomy motivation (Deci and Ryan, 2008; Ryan and Deci, 2000).

The use of SDT in education has been used to explain the role of the social environment and interpersonal interactions in promoting intrinsic motivation in students and teachers, and that needy support that meets students’ basic psychological needs will positively affect students’ motivation, engagement, and emotions, and that students will develop stronger intrinsic motivation, higher classroom engagement, and produce more positive emotional experiences when their needs are met (Trigueros et al., 2023; Wild et al., 2023; González-Arias et al., 2025). SDT also explains important factors and outcomes that influence the motivational development of pre-service and in-service teachers, and that support for pre-service and in-service teachers would be beneficial in promoting their basic psychological needs of a sense of autonomy, competence, and connectedness, which would in turn lead to autonomous motivation and thus increase the teachers’ sense of self-efficacy and satisfaction with their jobs (Guo and Xu, 2024; Kaplan, 2022). Studies have also utilized SDT to explain the relationship between pre-service teachers’ motivation and effective teaching; when student teachers have higher autonomous motivation, they perform better in terms of effective teaching (Burgueño et al., 2021; Teraoka et al., 2025;).

In summary, existing research within the Self-Determination Theory (SDT) framework has demonstrated that social environmental support promotes psychological well-being by satisfying individuals’ needs for competence, autonomy, and relatedness, thereby fostering intrinsic motivation. However, further investigation is needed to understand how social environmental support specifically facilitates the fulfillment of these competence, autonomy, and relatedness needs.

### Professional development and psychological needs of student teachers

Practicing teachers face numerous challenges and difficulties during their internships, students perceive that they are not noticed and valued in their internship schools and that their internship mentors do not show them enough acceptance and respect, and they also face pressures related to work survival such as adapting to the work environment, integrating into their work circles, and linking theory to practice, which can lead to the student teacher leaving the profession or career (Bloomfield, 2010). When student teachers enter the workplace, they have the need to be safe in the internship environment, the social need to establish good relationships with others, the need for self-affirmation to be able to utilize their abilities, and the need for self-fulfillment to be successful in the internship (Dreer, 2020).

And the psychological needs of student teachers during internships are often related to the challenges encountered during the internship process, where student teachers are faced with numerous pressures and are inhibited by their needs for competence, autonomy, and connection during the internship. Firstly, when student teachers are new to the internship school, they tend to encounter difficulties in applying their professional knowledge and skills to effectively carry out teaching activities, and thus feel that they do not have the ability to control their classrooms, and their sense of competence needs will not be met (Kaplan, 2021); secondly, when the internship school restricts the interns’ use of innovative pedagogies to a certain extent, it will inhibit the student teachers’ sense of autonomy (Huu Nghia and Tai, 2019); However, if the work environment is highly controlling and non-recognizable to the teacher it can lead to unmet feelings of autonomy for the student teacher, which prevents the teacher from focusing on developing students as well as creating more emotional exhaustion in the workplace (Fernet et al., 2016). And finally, student teachers who are not fully accepted by the internship school during their internships, are not given the same status as in-service teachers, and have difficulty accessing the need for connection when confronted with unfamiliar school environments and school cultures (Dreer, 2020).

In contrast, the fulfillment of student teachers’ autonomy, competence, and need for connection will benefit their professional development and teaching performance. When a sense of autonomy is met, student teachers experience a sense of authenticity that enhances the subjective experience of student teachers in teaching, they have room for their own ideas and choices, and a strong sense of personal and professional development (Heikonen et al., 2020; Romero et al., 2023). It also produces greater levels of self-efficacy and self-reflection (Dreer, 2020; Liu and Sitoe, 2019). Korthagen and Evelein (2016) suggest that student teachers whose needs are met produce more positive teaching behaviors. School and mentor support can help student teachers adapt to school culture, strengthen their overall sense of connection with colleagues and students, thereby enhancing their job satisfaction and intrinsic motivation for teaching (Kaplan et al., 2021). Unbiased, constructive feedback and encouragement can support student teachers’ need for a sense of competence, driving them to better implement and refine teaching practices while increasing work efficiency (Brenner, 2022).

### Role of internship mentors in supporting student teachers’ development

Teaching internship refers to an educational approach where education majors undertake practical training at relevant workplaces for a period to earn academic credits. Some countries refer to this as “teaching practicum,” “student teaching,” or “teaching practice” (Cai et al., 2022). Teaching internship prepares student teachers for their future teaching career by giving them first-hand experience of the teaching profession, and is a critical period for student teachers to construct their professional identity as teachers, which has a significant impact on their professional identity, development of professional knowledge and competence, and willingness to teach (Liu et al., 2025; Zhu et al., 2020), and the role of internship mentors is to help the student teachers integrate into the teaching team and help students familiarize themselves with the various rules and regulations of the school, and mentors should not only support interns in terms of professional knowledge and ability, but more importantly, provide emotional support for interns (Schechter and Orly, 2014). However, internship mentors often do not have the time and energy to mentor student teachers due to their busy work schedules and do not lead student teachers to integrate into the work environment and culture of their internships. Some internship mentors are even unable to accept and recognize student teachers’ new ideas, so student teachers do not receive emotional support such as recognition from the internship mentor and other teachers, in addition to not receiving the constructive advice that internship mentors should give (Rippon and Martin, 2006). However, gaining acceptance and recognition from the internship school and establishing a good relationship with the internship mentor is one of the most important sources for student teachers to feel a sense of belonging, and effective guidance and positive evaluation from the internship mentor can also help to alleviate the stress of the student teachers in the internship, enhance the sense of competence of the internship teachers, and play an important role in their career choices and the construction of their teacher identities (Caires et al., 2012; Hu et al., 2023; Zhang et al., 2024).

In summary, the needs support of mentors will fulfill the psychological needs of student teachers in internships and can relieve student teachers’ internship stress and dilemmas, enabling students to enhance their professional identities and firm up their career choices. Particularly against the backdrop of low social recognition, heavy workloads, low wages for preschool teachers, and China’s declining birth rate, the challenges for student teachers in adapting to the preschool teaching profession have intensified, making their professional identity formation even more challenging (Huang et al., 2022). Evidently, fulfilling psychological needs for autonomy, competence, and relatedness fosters professional identity among preschool teachers, mitigating attrition rates. This identity may indirectly influence outcomes through mediating effects on intrinsic motivation (Wong and Liu, 2024).

### Application of BPNT in Asia and the Chinese sociocultural context

BPNT has also been validated and applied in Asian countries to enhance teachers’ teaching motivation and job satisfaction. Guo and Xu (2024) examined the influence of Chinese student teachers’ satisfaction with basic psychological needs on teaching motivation, along with the mediating roles of teaching self-efficacy and emotions. Aiyoob et al. (2025) investigated the mediating role of Singaporean teachers’ satisfaction with basic psychological needs in the relationship between perceived school leadership support and teachers’ technology use and job satisfaction, finding that perceived school leadership support predicted teachers’ satisfaction with basic psychological needs.

China’s student teachers are profoundly influenced by Confucian culture, collectivist values, and exam-oriented education (Zhou et al., 2019). First, Chinese society prioritizes collective interests and emphasizes respect for teachers and learning, leading students to value interpersonal connections and hierarchical teacher-student relationships. Consequently, intrinsic motivation is easily driven by external environmental factors (He et al., 2022). Second, Confucian culture promotes a harmonious atmosphere where mentors feel responsible for caring for students. When evaluating pre-service teachers, mentors typically avoid direct criticism, instead expressing opinions indirectly through various linguistic modifiers (Ao et al., 2024). Third, traditional Chinese culture fosters a high-control leadership and mentoring style, frequently excluding student teachers from decision-making while prescribing goals, tasks, and organizational processes. Student teachers often choose to respect authority and comply with superiors (Kaplan et al., 2022). Additionally, Chinese preschools typically staff three early childhood educators per class, fostering hierarchical power structures among teachers. This complicates the circumstances faced by Chinese preschool education students during teaching practicums. Given China’s cultural context and the unique characteristics of its preschool education system, while some studies have explored the applicability of Self-Determination Theory (SDT) in Asian cultural settings for student teacher education, it remains necessary to utilize the psychological lens of SDT to uncover the psychological needs of Chinese preschool education students during practicums and the role of mentor support.

### Research gaps and questions

However, in previous research findings, Chinese scholars have paid less attention to student teachers and internship mentors in teaching practicums, particularly in creatively applying Western theories to the Chinese context to deeply explore the interaction process between mentors and student teachers (Yan and He, 2021). Domestic and foreign scholars have conducted fewer studies on the need fulfillment of internship teachers, as well as fewer studies on the relationship between student teachers and internship mentors from the perspective of SDT (Kaplan, 2022; Liu and Sitoe, 2019), and although more studies abroad have explored the psychological need fulfillment of student teachers based on SDT, more studies have used quantitative research and fewer have used qualitative research methods to explore the psychological needs of student teachers. Whereas the basic needs theory of SDT provides researchers with a reasonable framework to conduct research on the psychological needs of student teachers in the field of early childhood education, and the applicability of SDT across cultures has been demonstrated (Kaplan and Madjar, 2017), therefore, this study will apply the basic needs theory of SDT as a theoretical framework and utilize qualitative research methods to explore the psychological needs fulfillment of Chinese early childhood student teachers during the internships. Specifically, this study seeks to address two research questions:

1. How do early childhood student teachers perceive their mentors’ support for autonomy, competence, and relatedness during teaching internships?
2. How do they experience the fulfillment of their psychological needs within the context of mentorship?

## Methodology

### Case study

This study employed a case study approach to examine how early childhood student teachers experience and interpret mentor support for their psychological needs of autonomy, competence, and relatedness during teaching internships. A case study is particularly appropriate in this situation because it can capture the intricate details of a phenomenon within its context (Ketokivi and Choi, 2014). This approach is helpful in situations where the relationship between a phenomenon and its context is ambiguous because in this case a mentor-student teacher relationship was sought in authentic classroom settings. This approach shifts the focus to the student teachers’ lived experiences, which helps to appreciate the extent to which mentorship influences the development of psychological needs and professional identity. Moreover, this approach enables an in-depth exploration of the dynamic and context-specific interactions between mentors and student teachers, highlighting how these relational and developmental aspects of mentorship influence student teachers’ overall experience (Guo, 2019).

### Participants

The participants in this study were nine senior undergraduate student teachers from an early childhood education program at a university in Shanghai. In China, early childhood education includes children from birth to 5 years old, with preschool education serving children aged three to five. Such institutions are called kindergartens; thus “preschool” and “kindergarten” will be used interchangeably in this article. Participants for this study were chosen using purposeful sampling to select individuals who can provide in-depth information relevant to the phenomenon under study (Creswell, 2014). Specifically, participants were required to have completed at least 3 months of their internship to ensure sufficient mentoring experience. They were in the final stage of their undergraduate early childhood education program, ensuring they were fully immersed in internship practice. Additional criteria included interning in public kindergartens, which are regarded as representative of mainstream early childhood education in China, and being assigned a dedicated mentor to provide continuous guidance during the internship. Seven of them chose to intern at public kindergartens located in different districts of Shanghai, while two interned in public kindergartens in their hometown of Zhejiang Province. Preschool teachers in Shanghai largely prefer public kindergartens as employers because these institutions tend to pay better and offer better working conditions. Moreover, within the public kindergarten system in China, there is a tiered system where model kindergartens are the highest level, followed by first-grade kindergartens, then second-grade kindergartens at the lowest level. The sample size was kept intentionally small so that the lived experiences of each student teacher were thoroughly examined within the mentor-student teacher relational framework. Pseudonyms were used to protect the participants’ identities. Demographics of the participants are presented in Table 1.

**Table 1:** Demographics of the participants.

Name	Gender	Internship kindergarten location	Kindergarten type	Mentors’ teaching experience	Mentors’ role
Feng	Female	Putuo, Shanghai	Public, Model	30 years	Head teacher
Gong	Female	Yangpu, Shanghai	Public, Model	14 years	Teaching and research group leader
Xu	Female	Xuhui, Shanghai	Public, Model	23 years	Head teacher
Zou	Female	Qingpu, Shanghai	Public, Model	15 years	Head teacher
Wang	Female	Jiading, Shanghai	Public, Second-grade	13 years	Head teacher, parent committee supervisor
Huang	Female	Ningbo, Zhejiang	Public, First-grade	10 years	Head teacher
Wu	Male	Songjiang, Shanghai	Public, First-grade	10 years	Teaching and research group leader
Shen	Female	Minhang, Shanghai	Public, First-grade	16 years	Head teacher
Kong	Female	Wenzhou, Zhejiang	Public, Second-grade	12 years	Head teacher

### Data collection

Data were collected through semi-structured interviews with the student teachers, providing rich and contextual data (DiCicco-Bloom and Crabtree, 2006). The main questions in the interview protocol were based on the student teachers’ interactions with their mentors and how these interactions impacted the fulfillment of their autonomy, competence, and relatedness needs. The follow-up questions were asked as needed to clarify or deepen responses. The main interview questions that correspond to the SDT basic needs framework are presented in Table 2.

**Table 2:** Main interview questions.

Dimensions	Sub-dimensions	Interview questions
Background	Kindergarten	1. Could you briefly introduce the kindergarten where you completed your internship? (e.g., type, level, location)
	Mentor	Could you describe your mentor? (e.g., gender, years of experience, position, expertise)
SDT basic needs	Autonomy support	In what ways did your mentor provide you with opportunities to make decisions in your work? How did this influence you?
		How did your mentor support your teaching over time? How did this affect you?
		In what ways did your mentor support your personal interests or strengths during your internship? What did it mean for you?
	Competence support	How did you experience your mentor’s recognition of your work? Describe a moment you felt capable. What feedback led to that feeling?
		Could you describe the challenging tasks your mentor assigned? What support did your mentor provide when you handled the challenges?
	Relatedness support	How did your mentor support you in building relationships with colleagues or peers? How did these experiences make you feel?
		Could you describe the problem-solving experiences in teamwork? How did it influence your sense of belonging?
		Beyond work-related support, did your mentor show care for your personal life? Can you share an example?
Overall reflection		Looking back on your internship, how do you see your mentor’s role in this process?

Each interview was conducted privately and held in a venue chosen by the interviewee. Each interview lasted from 45 to 60 min. They were audio-recorded and transcribed verbatim. Data collection continued until sufficient depth and variation emerged across participants’ accounts. Analysis revealed no new substantial themes arising from the later interviews, indicating thematic saturation (Saunders et al., 2018). This saturation point ensured that the data was adequate to encompass the breadth of student teachers’ experiences in the study context. All participants gave their informed consent for the recording and use of their responses.

### Data analysis

The interview data were analyzed using thematic analysis to identify and interpret patterns or themes within qualitative data. Thematic analysis was chosen because it is robust and flexible enough to capture a breadth of data (Lochmiller, 2021). The lead researcher first read the transcripts of the interviews multiple times, taking note of repeating ideas and gaining a perspective on the overall narrative within the text. Three basic psychological needs of autonomy, competence, and relatedness, framed by SDT, served as the primary deductive, theory-driven coding scheme, but analytic flexibility was preserved by permitting inductive, data-driven codes to emerge within each domain. This anchored the analytic process both in the conceptual structure of the study and in the lived experiences reported by participants. Initial codes were then identified based on relevant text segments pertaining to the autonomy, competence, and relatedness needs. At the same time, peer coding was conducted to improve credibility of the analysis (Miles et al., 2019). Specifically, a colleague familiar with qualitative research independently coded a subset of the interview transcripts. Then the two sets of codes were compared, and any discrepancies were discussed until consensus was reached. Cohen’s Kappa was not calculated because both deductive and inductive coding were conducted. Inductive codes were flexible, emergent, and sometimes overlapping, so they were not suitable for a strict statistical measure of agreement. Instead, peer coding centered on discussion and consensus to ensure credibility, without necessarily quantifying the degree of agreement between the coders (O’Connor and Joffe, 2020). Clusters of codes produced the following associations: the codes such as “decision-making freedom,” “gradual delegation of responsibility,” and “personalized task assignment” all converged on the higher-order construct of autonomy; the codes such as “constructive feedback” and “challenging tasks” aggregated under competence; finally, the codes like “emotional care” and “team contribution” grouped around the concept of relatedness. Next, these codes were grouped into potential overarching themes and sub-themes that represented the core aspects of the mentoring experiences of the student teachers. These themes were then reviewed, revised and finalized to ensure that they accurately and comprehensively represented the data. Finally, the illustrative quotes from the participants were used alongside thematic conclusions so that the analysis could remain aligned with the research questions. This approach ensured the data analysis was systematic, transparent, and directly connected to the research aims.

Furthermore, member checking was used to ensure trustworthiness and improve the validity of the findings by allowing participants to examine the results. After formulating the themes, participants were given a brief overview of the major findings based on the interviews. This helped them to review and critique the findings and participate in the interpretation of the data. In addition, reflexivity was self-regulated during the analysis as it is an approach to ensure qualitative research rigor (Darawsheh, 2014).

## Results

**Figure 1:**
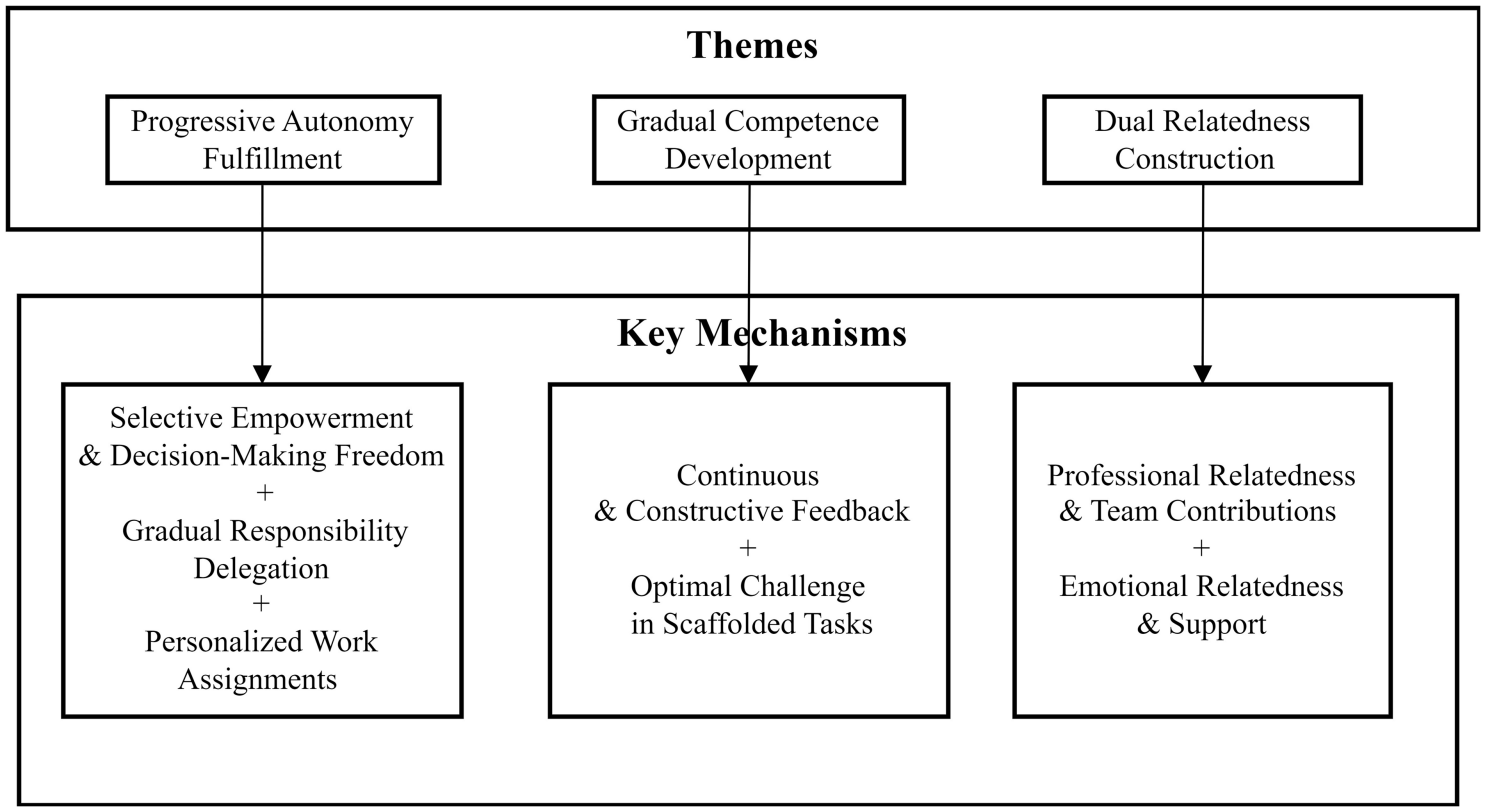
Themes and key mechanisms based on SDT framework.

From the participants’ narratives, three overarching themes emerged: progressive fulfillment of autonomy needs, gradual development of competence needs, and dual construction of relatedness needs. More specifically, these results show how autonomy is progressively nurtured through selective empowerment, gradual delegation of instructional responsibility, and personalized work assignments tailored to interests and strengths; competence is enhanced through constructive feedback and optimal challenge in scaffolded tasks; and relatedness is developed through emotional care and support, as well as contributions to teams. The following sections illustrate how these needs were satisfied within the context of the internship process and the influence they had on the student teachers’ professional development. Themes and key mechanisms based on the SDT framework are presented in Figure 1.

### Progressive fulfillment of autonomy needs

#### Selective empowerment and decision-making freedom

Many student teachers observed that although the overall structure of their tasks was framed by their mentors, mentors gradually released decision-making authority, granting limited but meaningful freedom in how to execute those tasks. This selective empowerment, where student teachers make choices within set boundaries, increased their engagement, creativity, and sense of agency. Feng shared: “My mentor allowed me to choose the style of the PPT as long as I completed the task on time. This gave me the opportunity to showcase my creativity.” She further clarified that such autonomy, even if it seemed trivial, increased her efficiency and investment towards her goals. Similarly, Xu remarked:

My tasks were mainly assigned by my mentor, but I had the opportunity to put forward some ideas and participate in discussions on some minor details like the design of the reading corner. This experience gave me a measure of agency which enhanced my motivation.

Wu noted that he was permitted to choose the reading materials for his literacy lesson: “I could pick the stories I used in class. This autonomy allowed me to align the content with my teaching style, which made the lesson feel more personal and engaging.” Likewise, Zou recounted being given the freedom to organize the game session of a physical activity: “I planned the game session independently and chose the music and structure by myself. That gave me a sense of being a real teacher, rather than just an assistant.”

#### Gradual delegation of instructional responsibility

This evolution from selective empowerment to gradual shift in instructional control marks a distinct developmental change: gradual delegation of instructional responsibility. As student teachers show readiness, mentors withdraw from their prescriptive guidance within the internship framework and gradually grant full control over lesson planning, organizing, and implementation. This shift reflects not only a growing sense of trust from mentors based on their assessment of the student teachers’ readiness, but also a transformation in the student teacher’s role from an executor to an initiator. Zou provided an example:

I participated in the teaching research meeting, where my mentor encouraged me to express my opinions. When my ideas were acknowledged, I felt that they were truly valued. Over time, I was trusted with designing and managing all game-based learning activities. My mentor granted me the autonomy to select and arrange tasks for the children, which gave me considerable flexibility in instructional planning.

Shen also experienced a gradual increase in instructional autonomy during her internship. Her mentor assessed her readiness for more responsibilities and independence in teaching. Shen recalled:

At the beginning, my mentor only allowed me to decide on the pedagogical strategies. As my teaching competence and confidence grew through practice, my mentor gradually granted me greater instructional responsibility, eventually entrusting me with full control over the design of an open class—including the thematic content, materials, and pedagogical approach. My mentor only gave suggestions when I actively sought assistance.

This contributed to her developing a strong sense of professional identity through taking ownership of instructional tasks. Reported similar experiences where his mentor gradually relinquished control based on her assessment of his readiness for more responsibilities. Initially closely supervised, he progressed to independently developing lesson plans: “I could choose the materials and design the activities on my own. I felt like a peer and not a student. This strengthened my pedagogical self-confidence, while simultaneously deepening my engagement with teaching.”

#### Personalized work assignments tailored to interests and strengths

Participants shared that mentors purposefully design work assignments that aligned with their interests, strengths, or past experiences. These personalized opportunities not only enhanced their engagement but also bolstered their self-efficacy and confidence. Wang’s mentor recognized her creative abilities and tasked her with sculpting the nature corner in the classroom using hand-made clay models. “This was something I enjoyed and was good at. Being able to use my creativity made me feel more confident,” she noted. Similarly, Gong recalled an experience where her passion for drawing was acknowledged. “My mentor remembered a casual conversation where I mentioned I liked to draw, and later gave me that project. It made me feel seen and valued.” she was assigned to illustrate a picture book cover, which was later displayed in the reading area. This experience turned her personal interest into a meaningful contribution.

Other participants shared comparable experiences. Kong was given the responsibility of leading the music activities because of her background in piano and dance, while Feng was tasked with designing the classroom environment because of her artistic strengths. As Feng stated, “I was responsible for painting wall decorations and designing the game corner, which allowed me to use my art skills.”

In summary, participants highlighted that mentors gradually fostered their autonomy by selectively granting decision-making freedom within structured tasks and progressively delegating full instructional responsibility based on assessment of their readiness. Personalized assignments tailored to their interests and strengths further enhanced engagement, creativity, and professional confidence.

### Gradual development of competence needs

#### Continuous and constructive feedback

Participants highlighted the importance of continuous and constructive feedback from mentors in developing their professional competence. While explicit praise was uncommon, they found that ongoing feedback and specific suggestions helped them recognize their progress and improve their instructional practice. Feng described her mentor’s feedback as a mirror that reflected both her strengths and areas for improvement. She explained: “Although my mentor rarely give me explicit praise, I could see my progress through her constructive feedback. She acknowledged the activities and classroom environment I had designed.” Wu also noted that although his my mentor rarely offered direct praise, she provided constructive advice during the design of classroom projects. He described how this iterative process of feedback and revision gradually improved his confidence and clarity in both classroom management and instructional planning. He explained: “Sometimes I made mistakes in how I explained activities or set up materials. My mentor would analyze my performance and point out where I was unclear and offer critical suggestions for improvement.”

Similarly, Gong underscored the value of repeated practice supported by critical and targeted feedback. As she stated, “After trying a few times, I really improved. These cycles of trials, feedback, and adjustment, guided by my mentor’s analysis of my teaching plans and classroom practice, cultivated my sense of instructional precision and flexibility.” In addition, Xu reflected on how her mentor’s indirect feedback functioned as internalized cues for self-regulation: “My mentor never praised me openly, but her comments like ‘this part needs rethinking’ or ‘how about trying another way?’ showed me areas where I had to change. Gradually, I started to anticipate feedback and adjust in advance.”

Likewise, Huang also shared that although her mentor’s feedback seemed harsh at first, it eventually broadened her understanding of the rationale behind instructional decisions. As she noted:

At first, I did not understand why my mentor was always so inquisitive about my plans. But later I came to understand those questions helped me think deeply and constructively. I started realizing teaching should not be viewed as a checklist but as something to constantly improve.

Her narrative reflects a cognitive shift from a task-oriented mindset to one focused on professional reflection and continuous improvement.

#### Optimal challenge in scaffolded tasks

Participants frequently emphasized that engaging with optimally challenging tasks assigned and scaffolded by mentors during their internships played a critical role in developing their teaching and classroom management skills. These tasks stretched their current skill levels, prompting them to acquire new strategies, adapt to dynamic classroom contexts, and build professional confidence. Kong was assigned by her mentor to lead a group teaching activity during her second month of internship, The most challenging part for her was managing the children’s behavior in group activities, especially how to handle unforeseen events. She recalled: “My mentor recommended some excellent online teaching videos for my learning and gave me tips beforehand. I was very glad that the group activity went more smoothly than expected. Gradually through practice, I learned how to resolve the issues.” Wu shared a comparable experience. He noted that large-group activities were often disruptive and initially left him feeling “overwhelmed, impatient and helpless.” He joked:

My mentor could read my mind. She encouraged me to bravely face my inner fears and take the challenges. She purposefully arranged me to lead large-group activities and asked me to observe and reflected on what she did in such activities. Through mentor scaffolding and repeated exposure, I gradually built confidence in managing these challenges.

In addition, Shen described the multifaceted challenges of independently leading an entire class session, a challenging task assigned by her mentor, which encompassed organizing the wrap-up activities and managing smooth classroom transitions. She recalled:

I had to make sure the kids were engaged, focused, and prepared for the next activity. It was hard to keep everything smooth, but this challenge provided a valuable opportunity to develop my teaching competence. My mentor connected me with a more experienced peer who had developed successful approaches. Through collaborative support, I learned to anticipate children’s needs and prepare in advance.

Wang articulated the technical and creative challenges involved in constructing a thematic learning space in her classroom. Her mentor asked her to design and build a clay bridge for the nature corner. She stated:

Achieving this goal required several iterations. I adjusted the design and tested for children’s safety. It pushed me to think practically and aesthetically at the same time. My mentor was very supportive. Whatever materials I needed, she bought or borrowed them for me. Completing this challenging task gave me a strong sense of accomplishment.

In summary, for these participants, continuous, constructive, and targeted feedback from mentors was instrumental in fostering their instructional competence, confidence, and reflective practice. In addition, the optimal challenge offered by their mentors, along with scaffolding provided in the form of resources, encouragement, and peer connections was critical in enabling them to achieve these challenges.

### Dual construction of relatedness needs

#### Professional relatedness and team contributions

Participants commonly reported that their sense of professional relatedness was significantly reinforced through active participation in team-based tasks and professional contributions. Engaging in these collaborative efforts allowed them to feel acknowledged, respected, and integrated into the kindergarten community. Wang described how involvement in both the design of the nature corner and suggesting improvements for classroom activities enhanced her perceived value within the teaching team. As she noted, “The recognition from my mentor and colleagues made me feel that I contributed to the team, which strengthened my determination to pursue a career in education.” Additionally, her mentor actively introduced her to other staff members and facilitated her interpersonal integration into the kindergarten community, further strengthening her professional relationships. Similarly, Zou stated: “My work helped improve the classroom environment and facilitate smooth class activities, which made me feel my importance in the team.” These experiences illustrate how concrete contributions and peer validation strengthened professional identity and fostered a deeper sense of belonging for the student teachers.

Wu echoed this sentiment, explaining that being responsible for the environmental layout for the entire grade and supporting communal kindergarten activities helped him feel “really part of the team.” He further noted that small acts such as assisting colleagues after work hours, strengthened his professional integration. Similarly, Shen’s technical support for colleagues and participation in large-scale public teaching events reinforced her team identity. She remarked: “Everyone recognized my efforts, and it made me feel respected and useful within the team.” Huang also noted how her creative contributions to thematic projects were acknowledged by others: “When it was finished, everyone praised it. It made me feel like my work really mattered.”

#### Emotional relatedness and support

Participants mentioned that their mentors started with a focus on guiding them through task completion, but gradually nurtured emotional support as the internship progressed. This emotional support was critical in strengthening student teachers’ sense of belonging, security and sustained motivation. As Feng stated, “Although my mentor’s attention was more on my work, there was some focus on my personal growth and emotional support. She gave me advice on how to schedule my university life and balance my academic and practical responsibilities.” Huang also remarked, “My mentor not only cares about my work tasks but also about my career development and future arrangements.”

Similarly, Wu recounted how his mentor extended care beyond professional obligations:

When I got sick, my mentor checked in on me, asked how I was doing, and told me not to push myself too hard. That made me feel really cared for, as if I wasn’t just an intern but part of the team. In addition, she offered career advice and often initiated casual conversations about my future, which deepened our emotional connection.

Emotional support also manifested in mentors’ responsiveness to personal struggles. Kong shared how her mentor offered her care and advice during a difficult period in her long-distance relationship: “That kind of attention made me feel being cared for.” Wang recounted how her mentor’s concern for her well-being: “She often reminded me to have a good rest after work. She once called me when I was late for work—not to scold, but to check if I was all right. It made me feel really included.”

In summary, involvement in team-based tasks and meaningful contributions strengthened the participants’ professional relatedness, fostering a sense of recognition, respect, and belonging within the kindergarten community. At the same time, mentors provided emotional support through personal attention and care, further enhancing the participants’ sense of security, inclusion, and sustained motivation.

## Discussion

### Autonomy: layered support via empowerment, delegation, and personalization

Autonomy is fulfilled through a layered and developmental process that involves progressive empowerment, gradual delegation of instructional responsibility, and personalized assignments tailored to student teachers’ interests and strengths. The study demonstrates that autonomy support starts from selective empowerment and evolves into deeper forms of decision-making as student teachers demonstrate readiness. The participant cases illustrate that task-level choices, even at the minimal level, fostered creativity and a sense of agency. This supports Ryan and Deci’s (2017) view that autonomy is grounded in some form of meaningful engagement, not merely absence of constraints. Furthermore, this layered approach demonstrates that autonomy in teaching practice is developmental, requiring mentors to continuously assess their developmental needs and readiness and respond to student teachers’ growing confidence and competence. By progressively scaffolding autonomy and providing personalized assignments, mentors supported meaningful engagement while fostering creativity, agency, and professional identity. This is in line with Reeve’s (2006) assertion that autonomy support is not the absence of structure but the variable provision of responsibility over time. Finally, personalized assignments tailored to the participants’ interests and strengths remained essential in deepening engagement. This aligns with Su and Reeve’s (2011) emphasis on adapting the learning context to the learners’ intrinsic motivations and illustrates how mentors can support autonomy through individualized acknowledgment rather than general encouragement.

This study finds that autonomy in the Chinese context operates within culturally defined limits as bounded autonomy. Mentors provided autonomy while negotiating decision-making within a set hierarchy of relationships and based on the readiness of the student teachers, which is consistent with Confucian norms that highlight the importance of authority and incremental trust (Lee et al., 2018; Wang et al., 2019). This form of bounded autonomy diverges from the SDT framework which states that autonomy is expressed through individual freedom of choice (Deci and Ryan, 2000). Therefore, the contextual reinterpretation of autonomy within SDT is necessary in authority-oriented cultures.

### Competence: skill and confidence building via feedback, challenge, and scaffolding

Competence support is a critical component of SDT. Competence needs are satisfied through providing positive feedback (oral praise) and optimal challenge (Ryan and Deci, 2017). Positive feedback helps students recognize their strengths and achievements, enhancing their sense of self-worth, and contributes positively to their confidence. However, the findings of this study extend this understanding by highlighting that participants valued constructive feedback over general positive reinforcement; this feedback was specific, actionable, and aimed at clarifying areas for improvement (Louca et al., 2022). Thus, while prior literature has emphasized the important role of praise in competence development (Ryan and Deci, 2017), participants in this study stressed that mentors’ constructive and critical feedback, not praise, was most effective in fostering professional development. This implies that competence support functions differently across cultures, with constructive and critical feedback working well as a motivator in contexts where rigor and high standards are valued (Chen, 2023). This aligns with Dweck’s (2006) assertion that strategically guiding students in recognizing their self strengths while pointing out areas that need refinement builds self-confidence and cultivates a growth mindset. This form of feedback is most effective when it is timely, specific, and developmentally appropriate, facilitating progress toward goals and encouraging sustained effort (Hattie and Timperley, 2007).

In addition to feedback, mentors’ provision of optimally challenging tasks was critical in facilitating student teachers’ development. This is consistent with Csikszentmihalyi’s (2014) concept of balancing challenge and skill. Importantly, these tasks were accompanied with appropriate scaffolding of mentors, enabling student teachers to expand their abilities through effortful engagement, build confidence, and sustain motivation.

### Relatedness: dual pathways to belonging and integration

In this study, when mentors cared for student teachers’ personal well-being, it enhanced their perceptions of emotional support and belonging. This supports Jevtić and Rogulj’s (2022) claim that mentors who express care and concern towards student teachers tend to create an emotionally supportive environment. Beyond emotional support, relatedness was also fostered through professional collaboration. In line with Helleve (2017) who argued that mentors facilitating collaboration fosters professional growth as well as professional identity, this study showed that mentors who encouraged collaboration and active participation helped students affiliate with their professional community and solidify their emerging professional identity. In addition, participants stressed the value of actively connecting students with other colleagues and professionals, assisting in the development of a supportive professional network. Such networks serve as resources for professional development and contribute to professional relatedness. These findings are in agreement with Bjorklund Jr et al. (2020) and Polizzi et al. (2021) who provided evidence that relational closeness within faculty learning communities enhances self-efficacy, demonstrating the competence and identity building function of professional networks.

Although SDT has identified relatedness as a basic psychological need, prior conceptualizations have largely emphasized interpersonal care, belonging, and team contribution (Ryan and Deci, 2017), providing only a general account of how relatedness is experienced. This study advances this understanding by proposing a dual framework of emotional relatedness and professional relatedness. The former is grounded in personal care and interpersonal warmth; the latter is anchored in collaboration, recognition, and contribution to shared goals within the community. These two distinct yet interconnected dimensions jointly shape how student teachers experience relatedness in mentorship.

Building on this dual framework, it is worth noting that SDT defines relatedness as the perception of being accepted and appreciated (Ryan and Deci, 2000), but this perception takes on a different form within the educational context of China. In this study, emotional relatedness is amplified by mentors’ concern for the participants’ personal well-being, echoing relational norms of care beyond professional boundaries. Simultaneously, professional relatedness is reinforced by collective goals and a strong sense of team affiliation, characteristic of collectivist values that prioritize group harmony and contribution (Bjorklund Jr et al., 2020; Zheng et al., 2020).

### Model synthesis and practical implication

**Figure 2:**
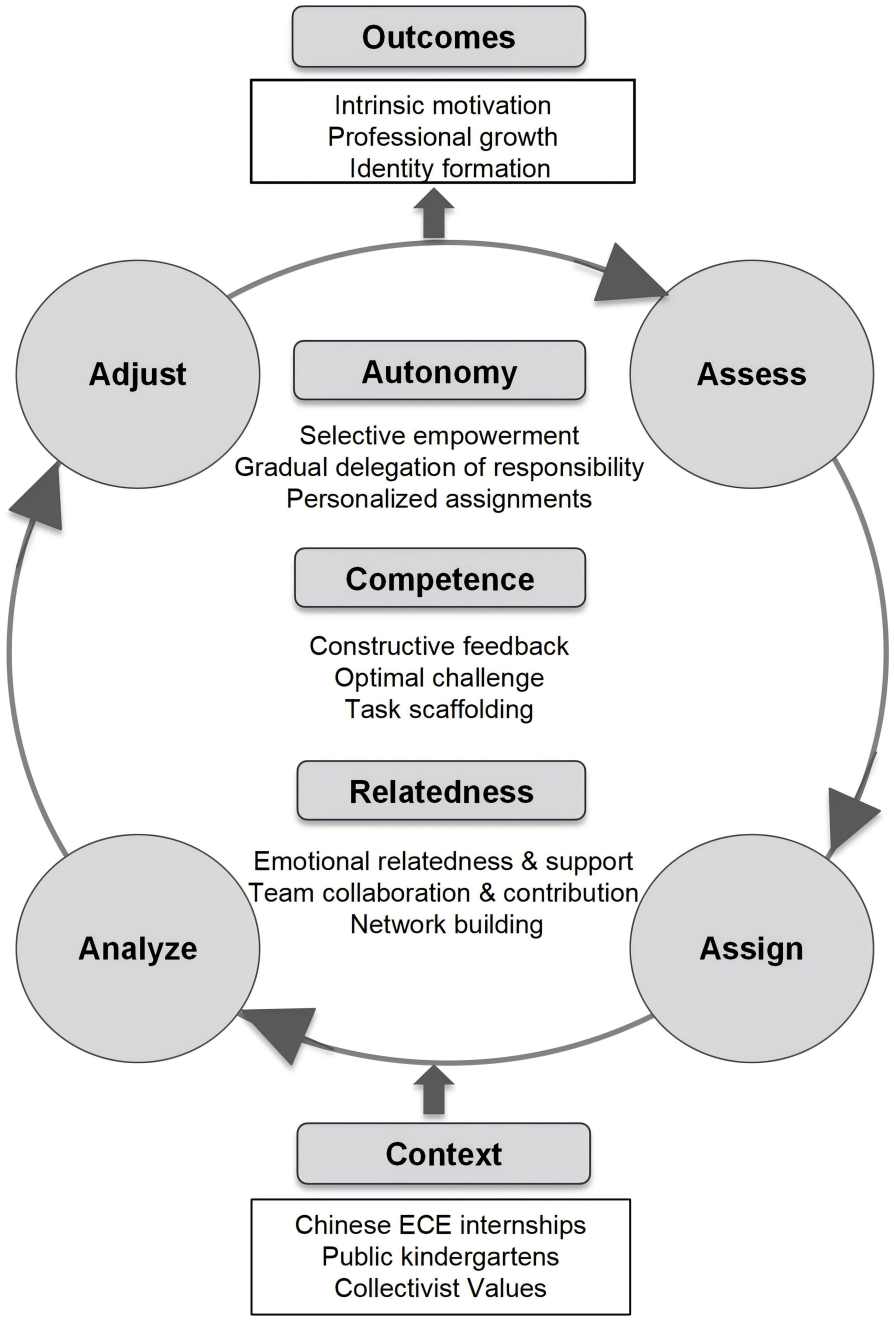
The developmentally responsive mentoring for motivation (DRMM) model.

The findings of this study can be synthesized into the Developmentally Responsive Mentoring for Motivation (DRMM) model in Figure 2. In the DRMM model, the calibration loop is illustrated as four stages: Assess, Assign, Analyze, and Adjust (4A). Mentors first assess student teachers’ readiness using their own mentoring experience, initial observations, as well as conversations with the student teachers. Then, based on this assessment, mentors assign tasks that are optimally challenging and scaffolded to stimulate student teachers’ development, and also take into account their personal interests and strengths. Next, mentors analyze the student teachers’ performance and provide them with specific, clear, and actionable feedback, highlighting effective strategies and identifying areas for improvement. Finally, mentors adjust their guidance by changing the level and type of support provided, including gradually increasing responsibility, refining support strategies, or strengthening relationships within professional communities. This iterative loop illustrates mentoring as dynamic and developmentally responsive.

The process is driven by three interrelated mechanisms. Autonomy support is granted through selective empowerment, gradual delegation of responsibility, and personalized assignments tailored to student teachers’ strengths and interests. Competence support is fostered through constructive feedback and optimally challenging tasks requiring efforts but are achievable through scaffolding. Relatedness support operates via emotional relatedness that concerns care of well-being, and professional relatedness that concerns collaboration, acknowledgment, and network building. Together, these contribute to intrinsic motivation, professional growth, and identity formation.

The DRMM model is designed to broaden existing mentoring scholarship by integrating autonomy, competence, and relatedness within a dynamic process that is both developmentally responsive and culturally situated. In Chinese public kindergartens, autonomy and relatedness are negotiated in accordance with collectivist values emphasizing bounded autonomy and team affiliation. This illustrates the cultural flexibility of the SDT needs.

At the same time, the DRMM model is not limited to the early childhood education sector or the Chinese context. Its core mechanisms include gradual delegation of responsibility, optimally challenging and scaffolded tasks, constructive feedback, and relational integration. These mentoring strategies are adaptable across cultural contexts and educational levels, where student teachers face comparable difficulties in meeting their basic psychological needs. Gradual delegation of responsibility reflects a developmental principle that applies to student teachers across systems during the transition from guided practice to independent teaching. Similarly, optimally challenging tasks, when combined with appropriate scaffolding, are essential for professional growth in varied contexts, as they balance challenge with support to build competence. In addition, constructive feedback has long been recognized as a powerful motivator of reflection and improvement, even though its form may vary culturally. Finally, relational integration through professional collaboration, contribution, and network building is critical to a sense of belonging and sustained motivation, regardless of cultural background.

The fundamental principles underlying these mechanisms are transferable, even though their implementation may vary depending on institutional norms and cultural values. Thus, the DRMM model could serve as a conceptual framework to capture context-specific characteristics as well as its global applicability to teacher education programs.

### Limitations and recommendations for future research

This study has its limitations. First, the small geographically constrained sample limits generalizability beyond public urban ECE institutions. Future research could focus on a more diverse sample including private kindergartens, as well as rural contexts, expanding to other types of institutions and regions to strengthen the generalizability of the findings. Second, the focus on student teachers’ perspectives omits mentors’ viewpoints and observational data, which leaves gaps related to the understanding of bidirectional mentorship dynamics. Future studies could incorporate mentor interviews and classroom observations to triangulate the enactment and perception of need support to enrich the depth of analysis. Third, this study does not analyze impacts of the mentorship in the long term. Future longitudinal studies could address this by tracking participants after graduation and analyze how early mentorship influences preschool teachers’ retention, professional identity, and sustained motivation over time.

## Conclusion

This study highlights the importance of mentoring in facilitating the fulfillment of student teachers’ psychological needs for autonomy, competence, and relatedness. Informed by the framework of SDT, the findings reveal that these needs are fulfilled through structured and relational mentorship. Furthermore, this study extends SDT’s application to the field of early childhood teacher education by synthesizing the findings into the DRMM model. Developmentally responsive mentorship that addresses these psychological needs strategically not only enhances student teachers’ intrinsic motivation and professional identity but also provides a transferable framework for teacher mentoring across diverse educational settings.

## Data Availability

The raw data supporting the conclusions of this article will be made available by the authors, without undue reservation.
